# P-363. Containing an Outbreak of Carbapenemase-producing Organisms in a Singapore Hospital

**DOI:** 10.1093/ofid/ofae631.564

**Published:** 2025-01-29

**Authors:** Darius Beh, Glorijoy Tan, Jia Qi Kum, Gabrielle Chia, Huiru Kui, Joanna Tan, Ying Wei Tang, Aung Hein Aung, Angela Chow, Bee Fong Poh, Brenda Ang

**Affiliations:** Tan Tock Seng Hospital, Singapore, Singapore; Tan Tock Seng Hospital, Singapore, Singapore; Tan Tock Seng Hospital, Singapore, Singapore; Tan Tock Seng Hospital, Singapore, Singapore; Tan Tock Seng Hospital, Singapore, Singapore; Tan Tock Seng Hospital, Singapore, Singapore; Tan Tock Seng Hospital, Singapore, Singapore; Tan Tock Seng Hospital, Singapore, Singapore; Tan Tock Seng Hospital, Singapore, Singapore; Tan Tock Seng Hospital, Singapore, Singapore; Tan Tock Seng Hospital, Singapore, Singapore

## Abstract

**Background:**

Tan Tock Seng Hospital is a 1700-bed academic teaching hospital in Singapore. Active surveillance using rapid PCR for the detection of asymptomatic carriage of carbapenemase-producing organisms (CPO) is routinely performed (Fig. 1). Where available, identified carriers are cohorted or isolated under contact precautions. We describe a hospital-wide multispecies OXA-48-like and NDM outbreak, findings from epidemiological investigations and the subsequent infection prevention and control (IPC) measures.

Existing and intensified CPO screening strategies

**Figure:**
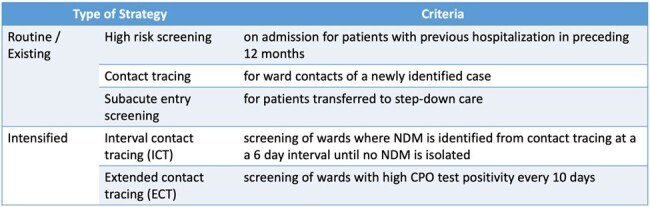

**Methods:**

Initial response included additional intensified contact tracing (Fig. 1), expansion of isolation bed capacity and audits of IPC practices. Case-control analysis involved 280 cases and 995 controls. Universal admission screening was conducted to validate the accuracy of existing high risk screening (HRS) criteria. Wards with high transmission underwent environmental sampling.

**Figure:**
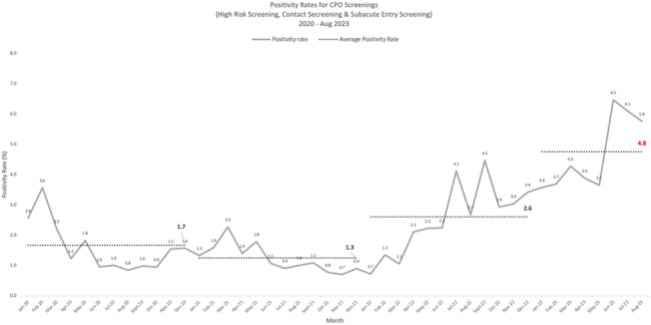

**Results:**

The CPO screening positivity rate was observed to be on a steady increase during 2022/23, from a baseline of 1.3-1.7% in 2020/21, peaking at 6.5% in June 2023 (Fig. 2). Spot audits demonstrated a decrease in hand hygiene compliance, improper cleaning of shared equipment, and poor IPC practices especially during diaper changes. Isolates were predominantly *Klebsiella pneumoniae* and sequence type 307. OXA was the commonest carbapenemase, followed by NDM. Risk factors for acquisition were presence of an indwelling urinary catheter for OXA (adjusted odds ratio [AOR] 1.62, 95% CI 1.02-2.48, p = 0.028), exposure to third-generation cephalosporins for NDM (AOR 3.83, 95% CI, 1.86-7.92, p < 0.001) and length of stay of 15-21 days for both (AOR 3.19, 95% CI, 2.05-4.96, p < 0.001). 4.8% of OXA carriers developed clinical infection within a median of 17 days (Fig. 3). Sensitivity of HRS criteria was 70%, though compliance to screening was only 60-80%. Periods of higher transmission were associated with lower rates of CPO cohorting (Fig. 4). CPO was found in 6.9% (9/130) of environmental samples.

Rate and time to onset of clinical infection for asymptomatic carriers according to CPO genotype

**Figure:**
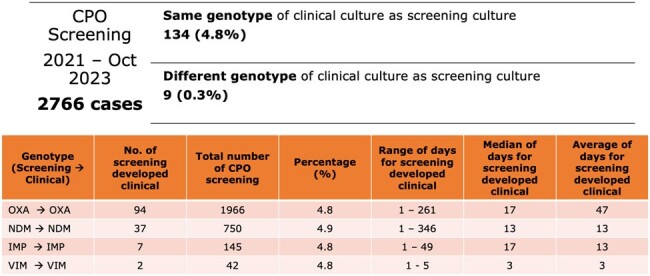

**Conclusion:**

Drivers of the outbreak were lower rates of CPO cohorting/isolation, a decrease in IPC standards, the inadequate compliance to HRS and the introduction of a highly transmissible bacterial clone. Early detection coupled with isolation remains a key strategy to control the spread of CPO.

Epidemiologic curve of the CPO outbreak in relation to cohorting efforts (%)

**Figure:**
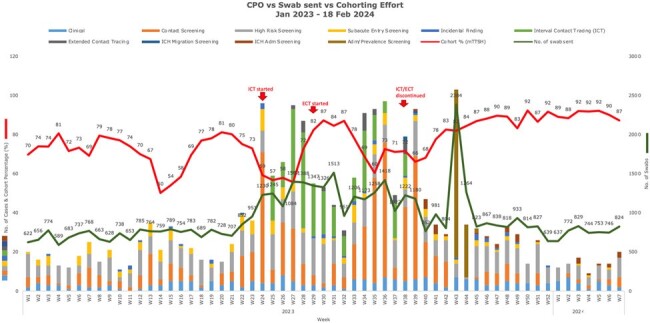

**Disclosures:**

**Darius Beh, MBBS (S'pore) MMed (Int Med) MRCP (UK)**, AstraZeneca: Conference Sponsorship|Gilead: Conference Sponsorship

